# Comparison of Robotic Surgery with Laparoscopy and Laparotomy for Treatment of Endometrial Cancer: A Meta-Analysis

**DOI:** 10.1371/journal.pone.0108361

**Published:** 2014-09-26

**Authors:** Longke Ran, Jing Jin, Yan Xu, Youquan Bu, Fangzhou Song

**Affiliations:** 1 Department of Bioinformatics, Chongqing Medical University, Chongqing, China; 2 Molecular Medicine and Cancer Research Center, Chongqing Medical University, Chongqing, China; 3 Department of Obstetrics and Gynecology, Southwest Hospital, Third Military Medical University, Chongqing, China; Baylor College of Medicine, United States of America

## Abstract

**Purpose:**

To compare the relative merits among robotic surgery, laparoscopy, and laparotomy for patients with endometrial cancer by conducting a meta-analysis.

**Methods:**

The MEDLINE, Embase, PubMed, Web of Science, and Cochrane Library databases were searched. Studies clearly documenting a comparison between robotic surgery and laparoscopy or between robotic surgery and laparotomy for endometrial cancer were selected. The outcome measures included operating time (OT), number of complications, length of hospital stay (LOHS), estimated blood loss (EBL), number of transfusions, total lymph nodes harvested (TLNH), and number of conversions. Pooled odds ratios and weighted mean differences with 95% confidence intervals were calculated using either a fixed-effects or random-effects model.

**Results:**

Twenty-two studies were included in the meta-analysis. These studies involved a total of 4420 patients, 3403 of whom underwent both robotic surgery and laparoscopy and 1017 of whom underwent both robotic surgery and laparotomy. The EBL (p = 0.01) and number of conversions (p = 0.0008) were significantly lower and the number of complications (p<0.0001) was significantly higher in robotic surgery than in laparoscopy. The OT, LOHS, number of transfusions, and TLNH showed no significant differences between robotic surgery and laparoscopy. The number of complications (p<0.00001), LOHS (p<0.00001), EBL (p<0.00001), and number of transfusions (p = 0.03) were significantly lower and the OT (p<0.00001) was significantly longer in robotic surgery than in laparotomy. The TLNH showed no significant difference between robotic surgery and laparotomy.

**Conclusions:**

Robotic surgery is generally safer and more reliable than laparoscopy and laparotomy for patients with endometrial cancer. Robotic surgery is associated with significantly lower EBL than both laparoscopy and laparotomy; fewer conversions but more complications than laparoscopy; and shorter LOHS, fewer complications, and fewer transfusions but a longer OT than laparoscopy. Further studies are required.

## Introduction

Endometrial carcinoma is the most common female genital tract malignancy in Western countries [1]. It is also the most common gynecologic cancer overall; 1 of every 40 women worldwide will develop endometrial cancer. Surgery is a major component of the diagnosis and treatment of endometrial cancer. Endometrial cancer is increasingly being treated with more minimally invasive approaches, including laparoscopy [2]. However, these minimally invasive approaches to the treatment of endometrial cancer have been limited due to long operation times (OTs), safety considerations, and other factors [3]–[5]. Thus, robotic surgery, the most novel minimally invasive technique, was developed to help overcome the technical limitations of laparoscopy and laparotomy. This technique enables surgeons to more easily perform complex procedures through improved visualization, more accurate instrument control, and improved ease of use of instruments [3]. Reza *et al.* [4] found that robot-assisted hysterectomy was associated with a longer OT but shorter length of hospital stay (LOHS), lower estimated blood loss (EBL), and fewer transfusions and complications than was open surgery. O'Neill *et al.* [5] showed that robot-assisted hysterectomy offers benefits with respect to a shorter LOHS and fewer blood transfusions than seen with open surgery.

However, the laparoscopic approach is reportedly a feasible alternative to conventional surgical treatment in patients with endometrial carcinoma [6]. Whether robotic surgery is superior to laparoscopy or laparotomy remains unclear. Therefore, the present meta-analysis compared the outcomes of the three currently used surgical approaches in patients with endometrial cancer: robotic surgery, laparoscopy, and laparotomy.

## Materials and Methods

### Literature search

The electronic databases of PubMed, MEDLINE, EMBASE, Web of Science, and the Cochrane Library were searched to identify eligible English-language publications (from January 1990 to September 2013). The following text and key words were used in the search: “laparoscopy and laparotomy,” “robotic-assisted with laparotomy,” “robotic versus open,” “robotic versus laparoscopic,” “robotics versus laparoscopy,” “robotics or laparoscopy,” “robotics and laparotomy,” “robotic versus laparotomy,” and “robotic-assisted laparoscopy, laparoscopy, and laparotomy” in combination with “endometrial cancer.” Logical combinations of these and related terms were used to maximize sensitivity. Finally, additional relevant articles were identified by searching the references of eligible articles.

### Selection criteria

The inclusion criteria for this meta-analysis were analysis of either a retrospective or prospective cohort; comparison of robotic surgery with laparoscopy or laparotomy for treatment of endometrial cancer; evaluation of the following six outcomes: OT, number of complications, LOHS, EBL, number of transfusions, total lymph nodes harvested (TLNH), and number of conversions; and clear documentation of the surgical techniques being compared (either “robotic” and “laparoscopy” or “robotic” and “laparotomy”). When the same institution reported more than one study, either the higher-quality or most recent publication was included in the analysis to avoid including the same patients.

The exclusion criteria for this meta-analysis were as follows: the above-mentioned outcomes of interest were not reported for the two techniques or it was impossible to calculate these outcomes from the published results, it was impossible to extract the appropriate data from the published results, neither the surgical outcomes nor patient parameters were clearly reported, and patients with endometrial cancer were not evaluated.

### Data extraction and quality assessment

Data extraction was carried out by two reviewers (L.R. and J.J.), and quality assessment was performed by another two reviewers (Y.X. and F.S.). Any disagreements were resolved by discussion between the reviewers. For continuous variables, the sample size, mean, and standard deviation (SD) were calculated. For dichotomous variables, the total number of patients in each group and the number of patients with each outcome of interest were calculated. Some studies reported median rather than mean values and range or interquartile range rather than SD; in such cases, the mean and SD were estimated [7]. Studies that gave no information on the SD or range were excluded from the meta-analysis. The study quality was assessed using the criteria developed by the Newcastle–Ottawa Scale (NOS) for quality assessment [8].

### Statistical analysis

The meta-analysis was performed using Review Manager (version 5.1.4; Copenhagen: The Nordic Cochrane Centre, The Cochrane Collaboration, 2008) and Stata (version 11.2; StataCorp LP; College Station, TX, USA) software. All test results were considered to be statistically significant at p<0.05. We analyzed dichotomous variables by estimating odds ratios with their 95% confidence interval (95% CI) and continuous variables using the weighted mean difference (WMD) with the 95% CI. The pooled effect was calculated using either a random-effects or fixed-effects model. Heterogeneity was evaluated with χ^2^ and I^2^ values. We considered significant heterogeneity to be present when χ^2^ was within the 10% level of significance (p<0.10) and the I^2^ statistic was>50%. If the I^2^ statistic was>50%, indicating significant heterogeneity, the random-effects model was used. Otherwise, the fixed-effects model was used. Possible publication bias was assessed by Begg's funnel plots and Egger's regression test.

## Results

**Figure 1:**
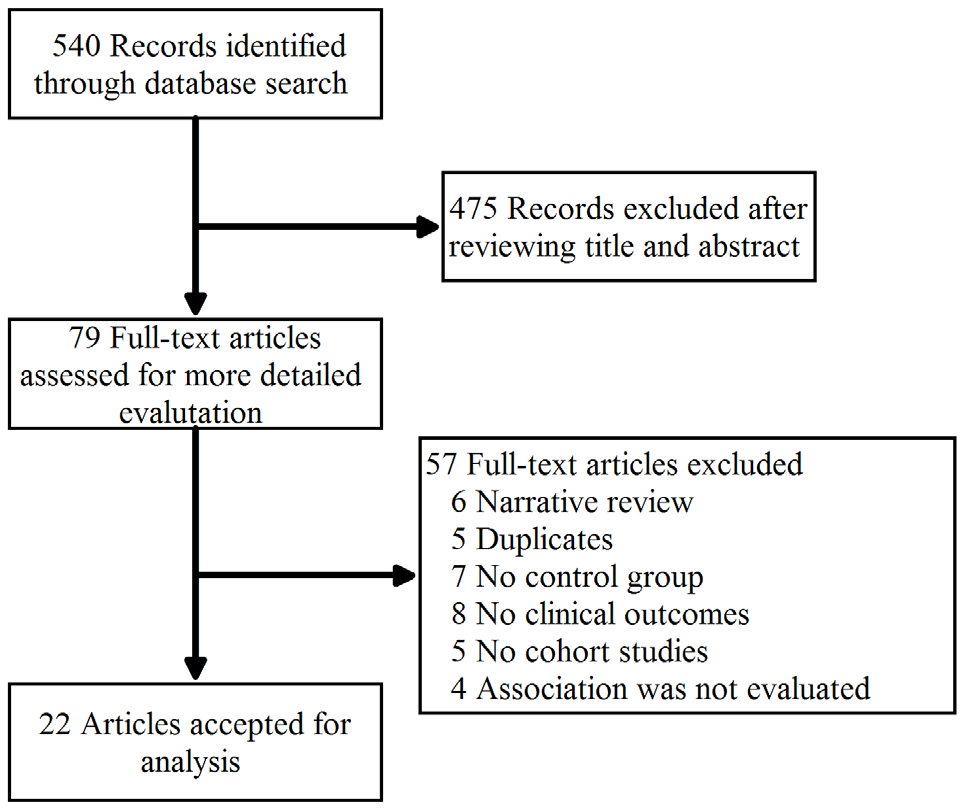
Flow diagram of identification of relevant studies in the present meta-analysis.

In total, 540 studies were identified and screened for retrieval using the above-described strategy. After screening the title or abstract, 475 studies were excluded and 79 were retrieved and evaluated in detail. Fifty-seven of these studies met the exclusion criteria, and 22 satisfied the selection criteria and were included in this meta-analysis (Fig. 1).

The patients' demographic and clinical characteristics are depicted in Table 1. The majority of studies (16/22, 72%) were carried out in the United States; there was one study each from Switzerland, Korea, Turkey, Spain, France, and Italy. Of all 22 studies, 8 compared robotic surgery and laparoscopy [9]–[16], 6 compared robotic surgery and laparotomy [17]–[22], and 8 compared robotic surgery, laparoscopy, and laparotomy [2], [19], [21], [23]–[27]. The 8 studies that compared robotic surgery and laparoscopy included 3403 participants (1822 who underwent robotic surgery and 1581 who underwent laparoscopy). The 6 studies that compared robotic surgery and laparotomy included 1017 participants (445 who underwent robotic surgery and 572 who underwent laparotomy).

**Table 1:** Patient demographic and clinical characteristics.

First Author, Year(Ref.#)	Study type	Country	No.of patients			Mean age(year)			Outcomes meased
			RS	LS	OS	RS	LS	OS	
Veljovich et al., 2008	Prospective cohort	USA	25	4	131	59.5	54	63	BMI,OT,EBL,LOHS,complications, uterine weight
Boggess et al., 2008	Prospective cohort	USA	108	81	138	61.9	62.0	64.0	BMI,OT,LOHS,conversion,EBL,total nodes,stage,complication,transfusion
Bell et al.,2008	Retrospective cohort	USA	40	30	40	63.0	68.4	72.3	BMI,OT,EBL,TLNH,Uterine weight,average cost,LOHS
DeNardis et al., 2008	Retrospective cohort	USA	56	-	106	58.9	-	6.5	BMI,FIGO stage,Grade,OT,EBL,LOHS,Transfusion rate,TLNH
Magrina et al., 2008	Prospective cohort	USA	27	31	35	50	31	35	BMI,OT,EBL, LOHS,FIGO stage,transfusion,readmission, TLNH
Seamon[1] et al.,2009	Prospective cohort	USA	32	17	14	55.0	52.8	42.0	BMI,FIGO stage,OT,EBL,TLNH,Surgical margins,Depth of invasion,complications,transfusions,LOHS,Follow-up,Survival status
Seamon[2] et al.,2009	Prospective cohort	USA	92	-	162	58	-	62	BMI,TLNH,conversion,transfusion,complications,OT,LOHS
Hoekstra1 et al.,2009	Prospective cohort	USA	32	7	26	62	59	56	BMI,Grade,Stage,OT,EBL,TLNH,LOHS,Conversion,Complications
Cardenas-Goicoechea et al.,2010	Retrospective cohort	USA	102	173	-	62	59.6	-	BMI,Tumor type,TLNH,Uterine weight,FIGO stage,Surgical time,Conversion,Blood transfusion,Estimated blood loss,LOHS
Sarlos et al., 2010	Prospective cohort	Switzerland	40	40	-	47	43.6	-	BMI,OT,EBL,Uterus weight,LOHS,Wound infection,Personnel costs
Jung et al.,2010	Prospective cohort	Korea	28	25	56	52.89	49.88	50.20	BMI,OT,Uterine weight,TLNH,Conversion, Overall complications,Transfusion,FIGO stage,LOHS
Göçmen et al., 2010	Prospective cohort	Turkey	10	-	12	55.7	-	56.4	BMI,EBL,Histology,FIGO stage,OT,conversions,LOHS,transfusion, complication,TLNH
Lim et al.,2010	Prospective cohort	USA	56	56	-	62.5	61.4	-	BMI,OT,EBL,TLNH,LOHS
Subramaniam et al., 2011	Retrospective cohort	USA	73	-	104	57	-	61.3	BMI,Grade,Uterine weight,TLNH,OT,EBL,LOHS,Wound complications,30 Day mortality
Goel et al.,2011	Retrospective clinical data	USA	59	-	38	66.5	-	59.5	BMI,OT,TLNH,EBL,Weight of uterus,FIGO stage,LOHS,Grade
Martino et al., 2011	Retrospective cohort	USA	101	114	-	61.8	63.6	-	BMI,Stage,TLNH,Total drug interventions
Coronado et al.,2012	Retrospective cohort	Spain	71	84	192	67.3	65.9	64.7	BMI,Grade,Stage,TLNH,OT,EBL,transfusions,conversion, complications,LOHS,Intra-operative
ElSahwi et al., 2012	Retrospective cohort	USA	155	-	150	62.4	-	65	BMI,LOHS,Stage,Grade,Uterine weight,TLNH,EBL,OT, Conversion
Venkat et al., 2012	Retrospective cohort	USA	27	27	-	58.2	60.2	-	BMI,Height,Weight,Hypertension,Smoker,TLNH,Uterine weight,LOSH,EBL,Stage,Grade,histology
Wright et al., 2012	Perspective	USA	1437	1027	-	-	-	-	Complication,Transfusion,LOHS,hospital cost
Seror et al.,2013	Retrospective cohort	France	40	106	-	66.27	66.91	-	BMI,Height,Weight,Hypertension,Hereditary history,OT,LOHS,Transfusion,FIGO stage,Complications
Fagotti et al., 2013	Retrospective case-controlled	Italy	19	38	-	62.0	61.9	-	BMI,OT,EBL,LOHS,complications

BMI(kg/m2):body mass index;OT(min):operating time; EBL(ml):estimated blood loss; FIGO: International Federation of Gynecology and Obstetrics;LOHS(h):length of hospital stay;TLNH(%): total lymph nodes harvested;RS:robotic surgery;LS: laparoscopy surgery;OS: open surgery;-:not available;

### Quality assessment

The present review evaluated no randomized controlled trials; all included studies were retrospective or prospective studies. The study characteristics and participant features are given in Table 1. The main characteristics of the 22 studies were assessed using the NOS. All studies scored moderately well on the NOS. A score of 7 was attained by eight studies [11], [13], [18], [19], [25]–[27], a score of 8 was attained by seven studies [10], [12], [14]–[16], [20], [22], and a score of 9 was attained by seven studies [2], [9], [17], [21], [23], [24], [28].

### Publication bias

**Figure 2:**
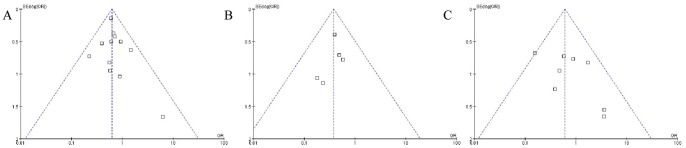
Funnel plot for main operative outcomes (complications, conversions, and transfusions) among all studies that compared robotic surgery and laparoscopy. (A) Publication bias regarding complications (Begg's test: Z = 0.48, p = 0.63; Egger's test: t = 1.03, p = 0.032). (B) Publication bias regarding conversions (Begg's test: Z = 1.22, p = 0.022; Egger's test: t =  −1.64, p = 0.20). (C) Publication bias regarding transfusions (Begg's test: Z = 0.62, p = 0.54; Egger's test: t = 1.55, p = 0.17).

Funnel plot analysis was performed for those studies that compared the numbers of overall complications, conversions, and transfusions between robotic surgery and laparoscopy. None of the studies lay outside the limits of the 95% CIs, and there was no evidence of publication bias among the studies (Fig. 2). The funnel plot for robotic surgery versus laparotomy showed no publication bias because there were no studies with a smaller mean difference (0–50) or higher variability (SE 16–20).

### Operative outcomes of robotic surgery versus laparoscopy

#### OT

**Figure 3:**
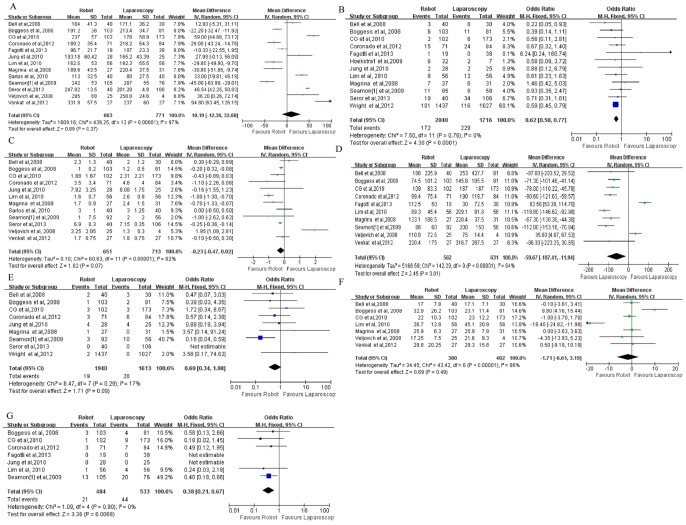
Comparison of robotic surgery and laparoscopy with respect to (A) operating time, (B) complications, (C) length of hospital stay, (D) estimated blood loss, (E) transfusions, (F) total number of lymph nodes harvested, and (G) conversions. OR: odds ratio; WMD: weighted mean difference.

Thirteen studies showed no statistically significant differences in OT between robotic surgery and laparoscopy. Analysis of the pooled results also showed that the two types of surgery did not significantly differ in this regard (WMD, 10.19; 95% CI, −12.30–32.68; p = 0.37) (Fig. 3A).

#### Complications

Twelve studies showed a significantly higher number of complications in robotic surgery than in laparoscopy. Analysis of the pooled results also showed that the number of complications was significantly lower in robotic surgery than in laparoscopy (OR, 0.62; 95% CI, 0.50–0.77; p<0.0001) (Fig. 3B).

#### LOHS

Twelve studies showed no significant difference in LOHS between robotic surgery and laparoscopy. Analysis of the pooled results also showed that the two types of surgery did not significantly differ in this regard (WMD, −0.23; 95% CI, −0.47–0.02; p = 0.07) (Fig. 3C).

#### EBL

Ten studies showed significantly lower EBL in robotic surgery than in laparoscopy. Analysis of the pooled results also showed significantly lower EBL in robotic surgery than in laparoscopy (WMD, −59.67; 95% CI, −107.41–11.94; p = 0.01) (Fig. 3D).

#### Transfusion

Nine studies showed no significant difference in the number of transfusions between robotic surgery and laparoscopy. Analysis of the pooled results also showed that the two types of surgery did not significantly differ in this regard (OR, 0.60; 95% CI, 0.34–1.08; p = 0.09) (Fig. 3E).

#### TLNH

Seven studies showed no significant difference in the TLNH between robotic surgery and laparoscopy. Analysis of the pooled results also showed that the two types of surgery did not significantly differ in this regard (WMD, −1.71; 95% CI, −6.61–3.19; p = 0.49) (Fig. 3F).

#### Conversions

Seven studies showed significantly fewer conversions in robotic surgery than in laparoscopy. Analysis of the pooled results also showed that the number of conversions was significantly lower in robotic surgery than in laparoscopy (OR, 0.38; 95% CI, 0.21–0.67; p = 0.0008) (Fig. 3G).

### Operative outcomes of robotic surgery versus laparotomy

#### OT

**Figure 4:**
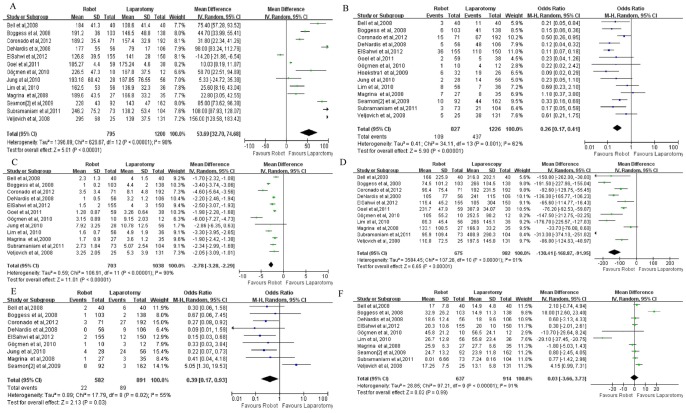
Comparison of robotic surgery and laparotomy with respect to (A) operating time, (B) complications, (C) length of hospital stay, (D) estimated blood loss, (E) transfusions, and (F) total number of lymph nodes harvested. OR: odds ratio; WMD: weighted mean difference.

Thirteen studies showed that the OT was significantly longer in robotic surgery than in laparotomy. Analysis of the pooled results also showed that the OT was significantly longer in robotic surgery than in laparotomy (WMD, 53.69; 95% CI, 32.7–74.68; p<0.00001) (Fig. 4A).

#### Complications

Fourteen studies showed significantly fewer complications in robotic surgery than in laparotomy. Analysis of the pooled results also showed significantly fewer complications in robotic surgery than in laparotomy (WMD, 0.26; 95% CI, 0.17–0.41; p<0.00001) (Fig. 4B).

#### LOHS

Twelve studies showed that the LOHS was significantly shorter in robotic surgery than in laparotomy. Analysis of the pooled results also showed that the LOHS was significantly shorter in robotic surgery than in laparotomy (WMD, −2.78; 95% CI, −3.28 to −2.29; p<0.00001) (Fig. 4C).

#### EBL

Eleven studies showed significantly lower EBL in robotic surgery than in laparotomy. Analysis of the pooled results also showed significantly lower EBL in robotic surgery than in laparotomy (WMD, −130.41; 95% CI, −168.87 to −91.95; p<0.00001) (Fig. 4D).

#### Transfusion

Nine studies showed significantly fewer transfusions in robotic surgery than in laparotomy. Analysis of the pooled results also showed significantly fewer transfusions in robotic surgery than in laparotomy (WMD, 0.39; 95% CI, 0.17–0.93; p = 0.03) (Fig. 4E).

#### TLNH

Ten studies showed no significant difference in TLNH between robotic surgery and laparotomy. Analysis of the pooled results also showed that the two types of surgery did not differ in this regard (WMD, 0.03; 95% CI, −3.66–3.73; p = 0.99) (Fig. 4F).

## Discussion

Surgical management has long been the primary therapy for endometrial cancer. Multiple surgical approaches to endometrial cancer have been available since newly developed methods of minimally invasive surgery were introduced. The choice of the most appropriate surgical method is becoming increasingly more important with growth in the obese and morbidly obese populations. To the best of our knowledge, this is the first comprehensive meta-analysis to compare robotic surgery with both laparoscopy and laparotomy for treatment of endometrial cancer. The results of this study show that robotic surgery is superior to laparotomy in terms of the number of complications, LOHS, EBL, and number of transfusions but is inferior to laparotomy in terms of the OT. Additionally, robotic surgery is superior to laparoscopy in terms of the EBL and number of conversions but is generally equivalent to laparoscopy in terms of the OT, number of complications, LOHS, and number of transfusions.

Robotic surgery may offer benefits over laparotomy in terms of reduced numbers of complications and transfusions, shorter LOHS, and lower EBL. The results of this study are consistent with those of previous studies [4]. In addition, the OT was longer in robotic surgery than in laparotomy. However, there was no significant difference in the TLNH between robotic surgery and laparoscopy. The numbers of conversions to laparotomy and complications are critical in minimally invasive surgical procedures for endometrial cancer. They are the most commonly reported outcomes because patients who have undergone conversion to laparotomy have higher complication rates [29]. The number of complications was significantly lower in robotic surgery than in laparoscopy. This result was due to both the inferior visualization of the laparoscopic videoscope and the superior ability of the three-dimensional robotic surgical platform, which enhances operative visualization [11]. Jung *et al.* [25] found that the high rate of operative complications among patients who underwent laparotomy was caused by the operative wound associated with the procedure.

In the present study, robotic surgery generally resulted in fewer conversions and lower EBL than did laparoscopy. These results can be explained by the robotic platform, which offers increased precision and dexterity, and are consistent with the results of previous studies [2], [23], [25], [29]. There were no significant differences in the OT or LOHS between robotic surgery and laparoscopy. Boggess *et al.* [2] and Jung *et al.* [25], however, found that robotic surgery was associated with a shorter LOHS and OT than was laparoscopy. Seamon *et al.* [30] noted that the OT is not consistently defined, making it difficult to compare this parameter between robotic surgery and laparoscopy in the face of heterogeneous data involving either a lack of the definition of OT in a given publication or the presence of data collection bias (retrospectively versus prospectively collected data). In another study [15], the OT included the time required to place the laparoscopic ports and uterine manipulator with colpotomy ring as well as the robotic docking time. However, other studies did not include these factors in the definition of OT. One possible explanation for the discrepant conclusions among various studies is the learning curve for robotic surgery in the treatment of endometrial cancer. Differences in the LOHS may be explained by differences in the medical insurance systems and cultures of the various countries in which each study was performed. In the present meta-analysis, the number of complications was significantly higher in laparoscopy than in robotic surgery; this may be associated with the OT and LOHS, neither of which showed a significant difference between robotic surgery and laparoscopy.

Our meta-analysis indicates that robotic surgery is associated with fewer complications than is laparotomy. This is similar to the findings of other investigators. Frigerio *et al.* [31] compared laparoscopy and laparotomy and found fewer postoperative complications among patients who underwent laparoscopic-assisted vaginal hysterectomy. Gil-Moreno *et al.* [32] compared laparoscopy and laparotomy and found that the amount of blood loss, number of blood transfusions required, and LOHS were significantly lower in the laparoscopic group; however, the OT was significantly longer. On the other hand, we found significantly more complications in robotic surgery than in laparoscopy. This is not completely different from the results of other investigations [17], [30]. Our data also showed a significantly lower EBL, shorter LOHS, and fewer transfusions in robotic surgery than in laparotomy. These findings are also not different from those of other investigations. Bell *et al.* [23] showed that the transfusion rate was not statistically different between robotic surgery and laparotomy. Finally, our study demonstrated that robotic surgery was associated with more complications than in laparoscopy but fewer complications than in laparotomy.

More prospective studies are needed to fully compare the effectiveness of robotic surgery with that of laparoscopy and laparotomy. The results of this study should be interpreted while taking its limitations into account. First, the data used for the WMD statistical analysis were median and range rather than mean and SD. The mean and SD must be estimated from the median and range, which may result in error or inaccuracy. Second, the learning curve is very important, especially for inexperienced surgeons, and affects the training curve for robotic surgery. As experience with robotic systems increases, the natural expectation is that the OT, LOHS, and transfusion rate will tend to decrease [24]. Finally, this meta-analysis was characterized by heterogeneity in the OT, LOHS, EBL, TLNH, and conversion rate because it was impossible to match the patient characteristics among all studies. Additionally, the random-effects model took between-study variation into consideration, which may have had a limited influence on the results.

In summary, this is the first meta-analysis to compare three conventional surgical approaches to endometrial cancer (robotic surgery, laparoscopy, and laparotomy) with respect to OT, complications, LOHS, EBL, transfusions, TLNH, and conversions. Overall, the present study has shown that robotic surgery is a feasible and promising method for the treatment of endometrial cancer compared with both laparoscopy and laparotomy. Some articles [13], [18], [19], [26] have reported that the costs associated with robotic surgery are higher than those associated with laparoscopy; however, we believe that robotic surgery can be a feasible alternative technique when robotic costs are reduced. No randomized controlled trials were available for inclusion in this study, which may have biased the interpretation of the results. Randomized controlled trials must be included in future studies to more fully assess the long-term results of this new technology in the field of endometrial cancer.

## Supporting Information

Checklist S1 **PRISMA Checklist.**
(DOC)
